# Experimental infections of sand flies and geckos with *Leishmania* (*Sauroleishmania*) *adleri* and *Leishmania* (*S.*) *hoogstraali*

**DOI:** 10.1186/s13071-022-05417-1

**Published:** 2022-08-11

**Authors:** Lucie Ticha, Jovana Sadlova, Paul Bates, Petr Volf

**Affiliations:** 1 Department of Parasitology, Faculty of Science, Charles University, Prague, Czech Republic; 2 Division of Biomedical and Life Sciences, Faculty of Health and Medicine, Lancaster University, Lancaster, UK

**Keywords:** *Sauroleishmania*, Sand flies, *Phlebotomus*, *Sergentomyia*, Geckos, Leishmaniasis

## Abstract

**Background:**

Species belonging to the subgenus *Sauroleishmania* are parasites of reptiles, and traditionally considered to be non-pathogenic to mammals. Knowledge of the development of these parasites in sand flies and their mechanism of transmission is currently lacking. The main aim of this study was to test the susceptibility of various sand fly species to infection by two *Sauroleishmania* species, focusing on the localization of parasites in the sand fly intestinal tract.

**Methods:**

The development of *Leishmania* (*Sauroleishmania* [*S*.]) *adleri* and *Leishmania* (*S*.) *hoogstraali* was studied in six sand fly species (*Phlebotomus orientalis*, *P. argentipes*, *P. sergenti*, *P. papatasi*, *P. duboscqi*, *Sergentomyia schwetzi*). Sand flies were fed through a chick-skin membrane on blood containing *Sauroleishmania* promastigotes, and they were dissected at various time intervals post blood meal (PBM). Guts were examined microscopically for the presence of parasites, and the intensity and localizations of infections were recorded. Morphological forms of both *Sauroleishmania* species developing in *P. orientalis* were analyzed. Experimental infections of geckos using sand fly-derived promastigotes were also performed, and the reptiles were repeatedly examined for *Sauroleishmania* infection by xenodiagnosis and PCR analysis.

**Results:**

High infection rates for both *Sauroleishmania* species were observed in *P. orientalis* and *P. argentipes*, with the parasites migrating anteriorly and undergoing a peripylarian type of development, including colonization of the stomodeal valve. Conversely, the development of *L*. (*S*.) *adleri* in *P. sergenti*, *P. papatasi* and *Se. schwetzi* was restricted to the sand fly hindgut (hypopylarian type of development). Five morphological forms were distinguished for both *Sauroleishmania* species developing in *P. orientalis*. All experimentally infected geckos scored negative for *Sauroleishmania* based on xenodiagnosis and molecular analysis.

**Conclusions:**

The results showed that *Sauroleishmania* promastigotes can undergo either a peripylarian or hypopylarian type of development in the sand fly intestinal tract, depending on the sand fly species infected. We demonstrated that *P. argentipes* and *P. orientalis*, two sand fly species known as permissive vectors for mammalian parasites of subgenus *Leishmania*, are also highly susceptible to *Sauroleishmania* as the parasites developed mature late-stage infections, including colonization of the sand fly stomodeal valve. Thus, the role of *Phlebotomus* sand flies in transmission of *Sauroleishmania* should be reconsidered and further investigated.

**Graphical Abstract:**

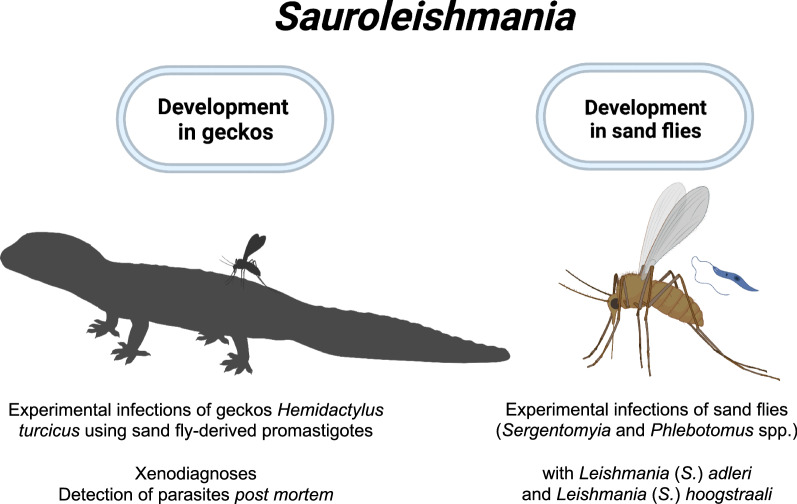

**Supplementary Information:**

The online version contains supplementary material available at 10.1186/s13071-022-05417-1.

## Background

Protozoa of the genus *Leishmania* (Kinetoplastida: Trypanosomatidae) are causative agents of leishmaniases. They are transmitted to vertebrate hosts by phlebotomine sand flies (Diptera: Psychodidae), with one exception, namely members of the subgenus *Mundinia*, for which biting midges (Diptera: Ceratopogonidae) are the main vectors [1]. The genus *Leishmania* is currently divided into four subgenera, of which three: *Leishmania*, *Viannia* and *Mundinia* include species infecting mammals, while the subgenus *Sauroleishmania* comprises of reptilian parasites [2, 3].

Various *Sauroleishmania* species have been found in reptiles of five families (Agamidae, Gekkonidae, Lacertidae, Scincidae and Varanidae) [4, 5], where they occur in two different forms: as free-living promastigotes and/or intracellular amastigotes [6]. Amastigote forms have been observed in different blood cells, mainly in monocytes or macrophages, but also in thrombocytes and erythrocytes [7–10].

The mechanism of *Sauroleishmania* transmission from sand flies to reptilian hosts is still being debated, as it has never been demonstrated under laboratory conditions [11]. Two possible modes of transmission are considered, either via sand fly bites and/or by ingestion of infected sand flies [6]. Sand flies of the genus *Sergentomyia*, which feed preferentially on cold-blooded animals, are generally accepted as the vectors of *Sauroleishmania* [12]. However, it has been reported that some *Sauroleishmania* species can cause late-stage infections in *Phlebotomus* sand flies; consequently, their possible involvement in *Sauroleishmania* transmission should also be considered [13, 14].

In the sand fly intestinal tract, the parasites initially multiply as promastigotes within the blood meal surrounded by a peritrophic matrix. Once the peritrophic matrix is broken, promastigotes migrate into various parts of the sand fly gut [14, 15] and, according to the description of Lainson and Shaw [16], undergo either hypopylarian, peripylarian or suprapylarian types of development. Hypopylarian development is confined to the hindgut (pylorus and rectum) and is considered typical of *Leishmania* (*Sauroleishmania* [*S*.]) species; peripylarian development includes a phase of development in the pylorus region of the hindgut followed by midgut and foregut colonization and is typical of *Leishmania* (*Viannia [V.]*) species, such as *L. braziliensis*; suprapylarian development occurs in the midgut and foregut only and is typical of *Leishmania* (*Leishmania* [*L*.]) species, such as *L. donovani*.

*Leishmania* (*S.*) *adleri* and *Leishmania* (*S.*) *hoogstraali* are two *Sauroleishmania* species distributed in sub-Saharan Africa. They were first isolated from the lacertid lizard *Latastia longicaudata* [17] and from the gecko *Hemidactylus turcicus* [18], respectively, but their vectors are unknown. The main aim of our study was to test the susceptibility of various sand fly species to *Sauroleishmania* infections. We investigated the development of *L.* (*S*.) *adleri* and *L.* (*S*.) *hoogstraali* in six sand fly species differing in susceptibility to *Leishmania*, with the focus on localization of parasites in the sand fly intestinal tract. We also performed experimental infections of geckos using sand fly-derived parasites.

## Methods

### Parasites, sand flies and geckos

Two *Sauroleishmania* species, *Leishmania* (*S*.) *adleri* (RLAT/KE/LV30) isolated from a lizard (*Latastia* sp.) in Kenya and *Leishmania* (*S*.) *hoogstraali* (RHEM/SD/LV31) isolated from a gecko (*Hemidactylus* sp.) in Sudan were used in this study. Cryopreserved parasites were shipped from Lancaster to Prague, and low-passage parasites (< 5) were used for the experimental infections of sand flies. Promastigotes were cultivated at 23 °C in Medium 199 (Sigma-Aldrich, Prague, Czech Republic) supplemented with 20% heat-inactivated fetal calf serum (Gibco, Prague, Czech Republic), 1% Basal Medium Eagle vitamins (Sigma-Aldrich, Prague, Czech Republic), 2% sterile human urine and 250 µl amikacin (Medopharm, Pozorice, Czech Republic).

In the first series of experiments, three sand fly species, each with a different susceptibility to various *Leishmania* species, were selected: *Sergentomyia schwetzi* (refractory to all *Leishmania* species tested so far [19]; colony originating from Ethiopia), and two *Phlebotomus* species: *Phlebotomus papatasi* (natural vector of *Leishmania major* [20]*,* colony originating from Turkey) and *Phlebotomus argentipes* (natural vector of *Leishmania donovani* [21]*,* colony originating from India). In the second series of experiments, we tested the susceptibility of three *Phlebotomus* species sharing an overlapping geographical distribution with the parasites used in the study: *Phlebotomus duboscqi* (colony originating from Senegal), *Phlebotomus sergenti* (colony originating from Turkey) and *Phlebotomus orientalis* (colony originating from Ethiopia). All sand fly colonies were maintained under standard conditions (26 °C, 14/10-h light/dark photoperiod, 50% sucrose), as described previously [22].

Twelve specimens of the gecko *Hemidactylus turcicus* were used for experimental infections with *Sauroleishmania* parasites. They were maintained individually in plastic boxes (32.5 × 22 × 21 cm) equipped with sand substrate, shelters and water dish, under a 12/12-h light/dark photoperiod and constant temperature maintained by heating pads and cables. Geckos were provided with water ad libitum, and twice a week they were fed with crickets (*Gryllus assimilis*) or mealworms (*Tenebrio molitor*) dusted in vitamins (Roboran, Univit, Czech Republic) to satiety.

### Experimental infections of sand flies

Fe﻿male s﻿﻿﻿and flies (5–9 days old) were experimentally infected by feeding through a chick-skin membrane on heat-inactivated blood containing 5 × 10^6^ promastigotes per milliliter. Engorged sand flies were then separated out, kept at 26 °C under standard conditions [22] and dissected at different time intervals post blood meal (PBM). The intensity of infections and localizations of parasites in the sand fly gut were examined under a light microscope. The intensity of infections was categorized according to Myskova et al. [23] as light/weak (< 100 parasites/gut), moderate (100–1000 parasites/gut) or heavy (> 1000 parasites per gut). All experiments were repeated at least twice for each *Sauroleishmania*-sand fly combination. Differences in infection rates were evaluated statistically by the Chi-square (*χ*^2^) tests using the SPSS version 27 statistical software package (SPSS IBM Corp., Armonk, NY, USA).

### Morphometry of parasites from gut smears

*Sauroleishmania* morphological forms were studied in *Phlebotomus orientalis* as this sand fly species was shown to be susceptible to both *L.* (*S*.) *adleri* and *L.* (*S*.) *hoogstraali*. Sand fly females were dissected on days 5, 7 and 9 PBM and their guts were used for analysis of morphological forms. Smears of sand fly guts positive for *Sauroleishmania* were fixed with methanol, stained with Giemsa staining solution and observed under a light microscope using an oil-immersion objective; promastigotes were photographed with an Olympus D70 camera (Olympus Corp., Tokyo, Japan). Body length, body width and flagella length of at least 140 randomly selected promastigotes from a minimum of three female sand flies were measured using ImageJ software and evaluated.

Morphological stages of the parasites were determined as described previously for members of the subgenus *Sauroleishmania* [14]: (i) long nectomonad promastigotes (body length ≥ 14 μm); (ii) short nectomonad promastigotes (body length < 14 μm and flagella length < 2-fold the body length); (iii) metacyclic-like promastigotes (body length < 14 μm and flagella length ≥ 2-fold body length); (iv) amastigote-like forms; and (v) haptomonad promastigotes. Differences in number of metacyclic-like stages were tested by the Chi-square (*χ*^2^) tests using SPSS software version 27 (SPSS IBM Corp.).

### Experimental infections of geckos

Geckos were infected with sand fly-derived parasites according to the methodology described for mammal-infecting *Leishmania* species [24] with a single modification: as the localization of metacyclic and reptile-infecting stages of *Sauroleishmania* have not been described yet, whole dissected sand fly guts (not only thoracic midguts) were used for the experimental infections. Briefly, two parasite-vector combinations displaying the highest infection rates and intensities of infections were chosen: *P. orientalis* for *L*. (*S*.) *adleri* and *P*. *argentipes* for *L*. (*S*.) *hoogstraali*. Sand fly females were infected by feeding through a chick-skin membrane with 10^7^ promastigotes per milliliter, as described in preceding text, and maintained under standard conditions until day 7 PBM. Engorged sand flies were then dissected and their guts examined for the presence of parasites under a light microscope. Sand fly guts with high parasite loads were pooled and homogenized in sterile saline solution. Each gecko was infected with 10 µl of homogenate, which corresponds to 10 sand fly guts.

Twelve geckos were separated into two groups of six specimens each for experiments with *L*. (*S*.) *adleri* and *L*. (*S*.) *hoogstraali*, respectively. Three geckos from each group were infected intraperitoneally by insulin syringe and the remaining three in each group were infected via the oral route using pipette tips. For each gecko infected via the oral route, 90 µl of saline solution was added to the sand fly gut homogenate. The infection doses (calculated using a Bürker chamber) were determined as 3.39 × 10^5^ for *L*. (*S*.) *adleri* and 7.5 × 10^4^ for *L*. (*S*.) *hoogstraali*.

Infected geckos were monitored weekly for the external signs of the infection, and they were examined for the presence of parasites at different time intervals post-infection (p.i.) using xenodiagnosis. At the end of the experiments (21 weeks p.i.) they were sacrificed and dissected. Samples from the liver, skin, tail, feet and blood were stored at − 20 °C for subsequent molecular analysis. Other parts of these tissues were cultivated on SNB-9 blood agar [25] with M199 medium as an overlay supplemented with 20% heat-inactivated fetal calf serum (Gibco), 1% Basal Medium Eagle vitamins (Sigma-Aldrich), 2% sterile human urine, 250 µl amikacin (Medopharm) and 1.5 µg/ml of fluorocytosin (Sigma-Aldrich). Cultures were checked microscopically for the presence of parasites once a week for a total of 5 weeks.

### Xenodiagnosis of geckos

Xenodiagnostic experiments were performed on weeks 3, 7, 12 and 18 p.i. using a laboratory-reared colony of *Se. schwetzi.* This was the only sand fly species which regularly fed on geckos in our laboratory. However, it does not support late-stage development of *Sauroleishmania* and parasites could be found in its midgut only before defecation. Each gecko was placed in separated net containing 30–50 sand fly females (5–7 days old) that were allowed to feed on the gecko for a maximum of 2 h. Engorged sand flies were separated out, maintained in the nets for 2 days and then (before defecation) placed individually into microcentrifuge tubes with Tissue Lysis Buffer (Roche, Prague, Czech Republic) and stored at − 20 °C for subsequent DNA extraction and analysis by PCR.

### PCR assay

Extraction of total DNA from sand flies and tissues of the geckos was performed using the High Pure PCR Template Preparation Kit (Roche Diagnostic, Mannheim, Germany) according to the manufacturer’s instructions. Extracted DNA was used as a template for PCR amplification targeting a region of the ribosomal internal transcribed spacer 1 (ITS1; approx. 300 bp) using the forward primer LITSR (5′-CTGGATCATTTTCCGATG-3′) and reverse primer L5.8S (5′-TGATACCACTTATCGCACTT-3′) as described previously [26]. Reactions were performed with EmeraldAmp® GT PCR Master Mix at the following cycling conditions: denaturation at 95 °C for 3 min; 35 amplification cycles of 95 °C for 20 s, 53 °C for 30 s, 72 °C for 40 s; and a final cycle at 72 °C for 6 min. The PCR products were analyzed using a SYBR Green fluorescent probe on 1% agarose gels. DNA extracted from the cultures of *Sauroleishmania* spp. and *Leishmania major* were used as positive controls (in the preliminary experiment, we tested these primers with various *Leishmania* species: *Leishmania* (*L.*) *major*, *Leishmania* (*L.*) *amazonensis* and *Leishmania* (*S.*) spp.; all of them gave positive results and, therefore, for the main experiment we chose only *L. major*). Additionally, serial dilutions were performed to confirm the detection of a minimum of 10^2^ parasites per sample.

## Results

### Experimental infections of sand flies I.

Development of *L.* (*S*.) *adleri* and *L.* (*S*.) *hoogstraali* was studied in *Se. schwetzi*, *P. papatasi* and *P. argentipes* at two time points: day 1 PBM (before defecation) and day 7 PBM (late-stage infection). Altogether, 506 female sand flies were dissected and examined for the presence of parasites. Statistically significant differences in infection rates were observed between the two *Sauroleishmania* species (Table 1).

**Table 1:** Comparison of infection rates of *Leishmania* (*Sauroleishmania* [*S*.]) *adleri* and *Leishmania* (*S.*) *hoogstraali* in three sand fly species

Sand fly species	Day 1 PBM	Day 7 PBM
*Sergentomyia schwetzi*	*χ*^2^ = 2.162, *df* = 1, *P* = 0.129	*χ*^2^ = 8.086, *df* = 1, *P* = 0.004
*Phlebotomus papatasi*	*χ*^2^ = 0.975, *df* = 1, *P* = 0.513	*χ*^2^ = 6.909, *df* = 1, *P* = 0.008
*Phlebotomus argentipes*	*χ*^2^ = 5.399, *df* = 1, *P* = 0.020	*χ*^2^ = 14.415, *df* = 1, *P* = < 0.001

Statistical analysis was performed using the Chi-square (*χ*^2^) test

*PBM* Post blood meal

**Fig. 1:**
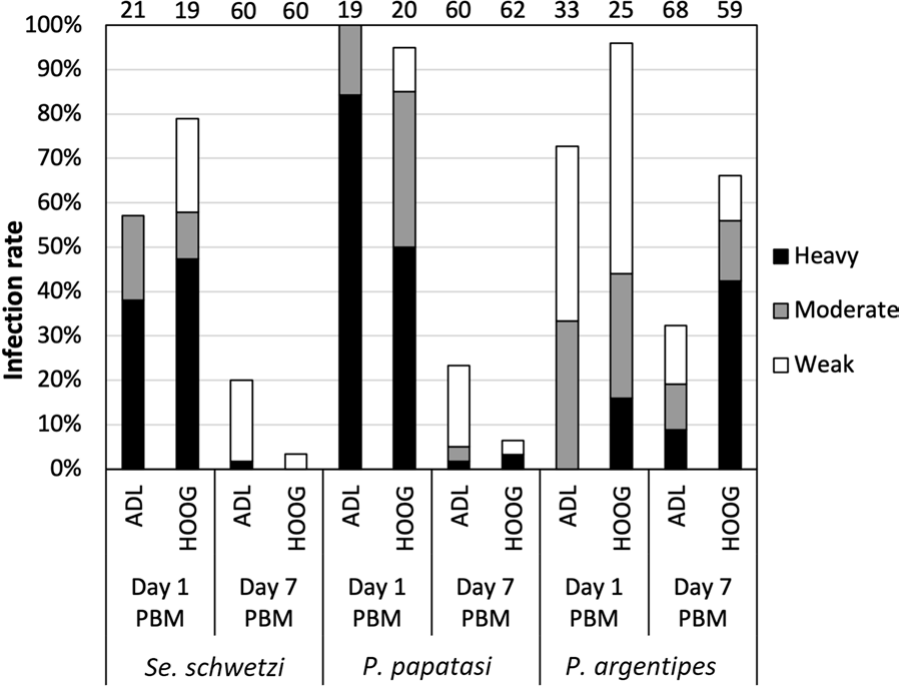
Development of *Leishmania* (*Sauroleishmania* [*S*.]) *adleri* and *Leishmania* (*S.*) *hoogstraali* in three sand fly species: *Sergentomyia schwetzi*, *Phlebotomus papatasi* and *Phlebotomus argentipes*. Infection rates were examined on days 1 and 7 post blood meal. The intensities of infections were categorized as heavy (> 1000 parasites/gut), moderate (100–1000 parasites/gut) or weak/light (< 100 parasites/gut). Number of dissected sand flies is indicated above the bars. Abbreviations: ADL *L.* (*S*.) *adleri*; HOOG, *L.* (*S*.) *hoogstraali*; PBM, post blood meal

On day 1 PBM, in all three sand fly species tested, variable but relatively high infection rates (57–100%) were observed for *L.* (*S*.)* adleri* and very high infection rates (79–96%) for *L*. (*S*.) *hoogstraali* (Fig. 1). All parasites were present in the ingested blood meal within the peritrophic matrix. Statistically significant differences in infection rates were observed on day 1 PBM among sand fly species tested for *L*. (*S*.) *adleri* (*χ*^2^ = 10.084, *df* = 2, *P* = 0.006), but not significant among sand fly species tested for *L*. (*S*.) *hoogstraali* (*χ*^2^ = 4.350, *df* = 2, *P* = 0.114).

**Fig. 2:**
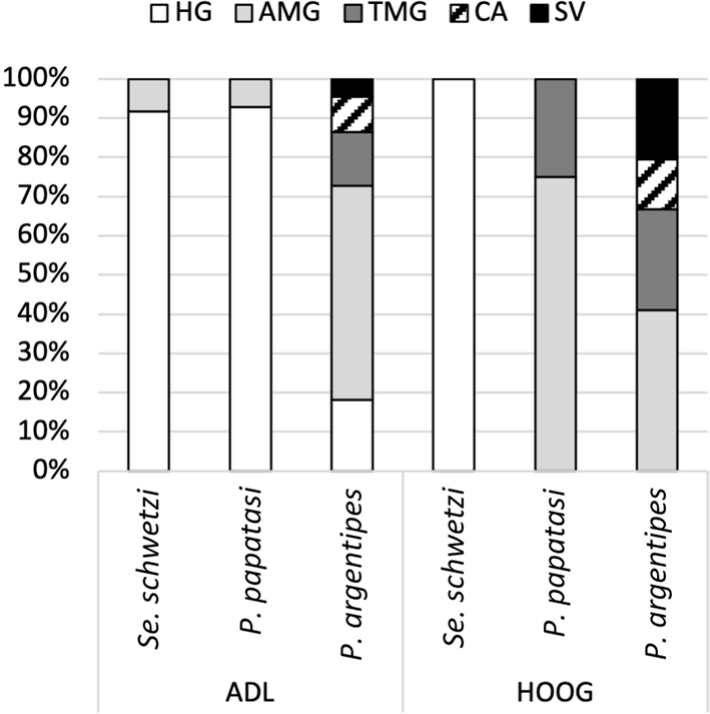
Simplified localization of *L.* (*S.*) *adleri* and *L.* (*S.*) *hoogstraali* in three sand fly species: *Se. schwetzi*, *P. papatasi* and *P. argentipes*. Localization of infections was evaluated microscopically on day 7 PBM. HG, hindgut; AMG, abdominal midgut; TMG, thoracic midgut; CA, cardia; SV, stomodeal valve. For more details, see Additional file 1: Table S1, Table S2

On day 7 PBM, after the defecation of blood meal remains, infection rates of both *Sauroleishmania* species were significantly reduced in *Se. schwetzi* and *P. papatasi. Leishmania* (*S*.) *adleri* was found in 20% of *Se. schwetzi* and 23% of *P. papatasi* females in which infections of weak intensity prevailed. Parasites occupied the hindgut (mainly pylorus and ileum) (Fig. 2), where attached haptomonad promastigotes were the prevailing forms, but in a few females, long free-swimming flagellates were also present. Infection rates of *L.* (*S*.) *hoogstraali* in *Se. schwetzi* and *P. papatasi* were negligible, reaching 4% and 7%, respectively.

In contrast, higher infection rates were observed in *P. argentipes* on day 7 PBM when 32% of dissected sand flies were positive for *L.* (*S*.) *adleri* and 66% for *L. *(*S*.)* hoogstraali*. *Leishmania* (*S*.) *adleri* developed in the hindgut, but also migrated anteriorly into the *P. argentipes* midgut (82% of infected sand flies). In two sand fly females, promastigotes reached the cardia (i.e. part of the midgut immediately behind the stomodeal valve), and in a single female the stomodeal valve was successfully colonized.

Infections of *L*. (*S*.) *hoogstraali* in *P. argentipes* were the most successful, with the presence of promastigotes detected in 66% of dissected sand flies on day 7 PBM. In most cases, parasites developed heavy-intensity infections and underwent the peripylarian type of development. In addition to the hindgut, promastigotes were observed in the abdominal and thoracic midgut (41% and 26%, respectively), reaching the cardia and colonizing the stomodeal valve in 13% and 21% of infected females, respectively.

Infection rates between sand fly species on day 7 PBM were not significantly different for *L*. (*S*.) *adleri* (*χ*^2^ = 2.782, *df* = 2, *P* = 0.249), but they were significantly different for *L*. (*S*.) *hoogstraali* (*χ*^2^ = 79.850, *df* = 2, *P*  ≤ 0.001).

### Experimental infections of sand flies II.

Development of *L.* (*S*.) *adleri* and *L.* (*S*.) *hoogstraali* was studied in *P. duboscqi*, *P. sergenti* and *P. orientalis* at various time intervals, namely on days 1, 5, 7 and 9 PBM. In total, 783 sand flies were examined for the presence of parasites.

#### Development of *L.* (*S*.) *adleri*

**Fig. 3:**
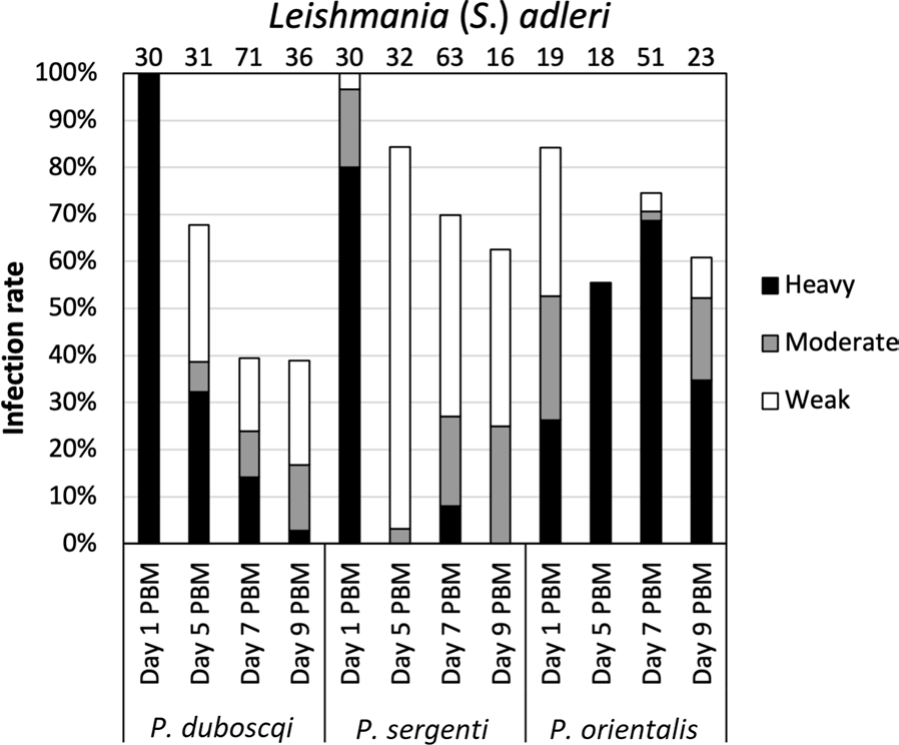
Development of *L.* (*S.*) *adleri* in three sand fly species: *Phlebotomus duboscqi*, *Phlebotomus sergenti* and *Phlebotomus orientalis*. Infection rates were examined on days 1, 5, 7 and 9 PBM. The intensities of infections were categorized as heavy (> 1000 parasites/gut), moderate (100–1000 parasites/gut) or weak/light (< 100 parasites/gut). The number of dissected sand flies is indicated above the bars

Promastigotes of *L.* (*S*.) *adleri* multiplied abundantly in the ingested blood meal on day 1 PBM, and infection rates reached 84–100% in all three sand fly species tested, with statistically significant differences among the three sand fly species (*χ*^2^ = 9.848, *df* = 2, *P* = 0.007; Fig. 3). Infections of heavy intensity prevailed in *P. duboscqi* and *P. sergenti*, while the intensity of infection in *P. orientalis* was slightly lower. In all tested sand flies, *L*. (*S*.) *adleri* successfully survived defecation and developed late-stage infections. Significant differences were found in infection rates among sand fly species on day 7 PBM (*χ*^2^ = 19.418, *df* = 2, *P* ≤ 0.001), while the differences were not significant on day 5 PBM (*χ*^2^ = 5.074, *df* = 2, *P* = 0.079) and day 9 PBM (*χ*^2^ = 3.852, *df* = 2, *P* = 0.146).

**Fig. 4:**
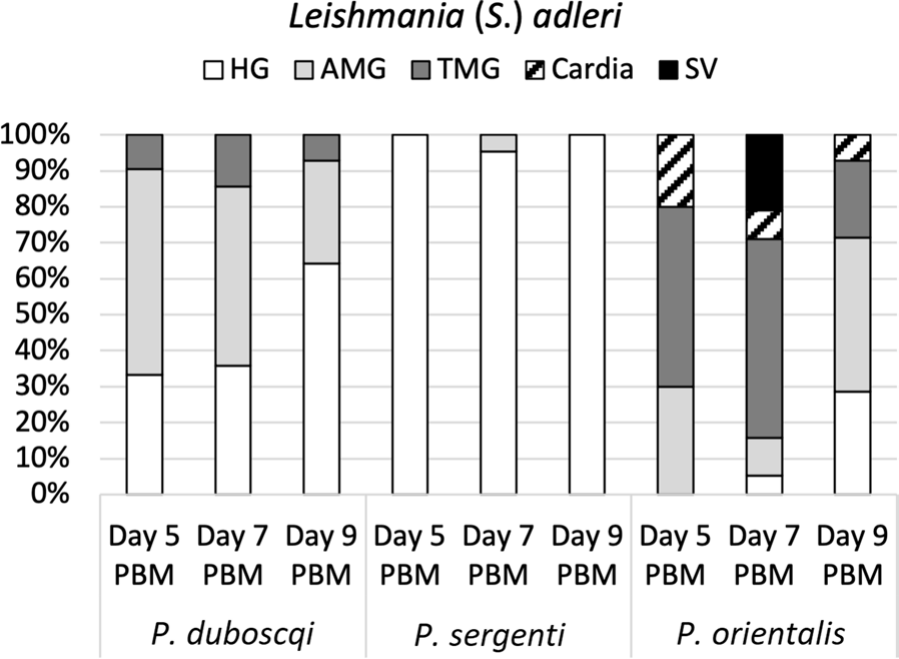
Simplified localization of *L.* (*S.*) *adleri* in three sand fly species: *P. duboscqi, P. sergenti* and *P. orientalis*. Localization of infections was evaluated microscopically on days 5, 7 and 9 PBM. For abbreviations, see Fig. 2. For more details, see Additional file 2: Table S3

In *P. duboscqi* the infection rate was almost 70% on day 5 PBM, then dropped to < 40% on days 7 and 9 PBM, with the majority of infections being of moderate and weak intensity. Promastigotes were localized in the hindgut and migrated to abdominal and thoracic midgut (peripylarian type of development) (Fig. 4). In the hindgut, haptomonad promastigotes were the most abundant forms, but free flagellates were also observed to a lesser extent.

Conversely, the hypopylarian type of development prevailed in *P. sergenti*. Relatively high infection rates were recorded at all designated time intervals (> 60%), and the intensity of most infections was moderate and weak/light. Parasites mainly occupied the hindgut (pylorus and ileum), with haptomonad promastigotes as the prevailing forms, while the presence of flagellates in the abdominal midgut was detected in only two *P. sergenti* females (5%).

In *P. orientalis,* heavy late-stage infections were observed on days 5 to 9 PBM, with > 50% positive sand flies, in which the peripylarian type of development prevailed. Promastigotes multiplied and migrated rapidly as they were present in thoracic midgut (50%) and cardia (20%) on day 5 PBM, and colonization of stomodeal valve had occurred in 21% of infected sand flies on day 7 PBM. Similar dynamics of the infections then persisted until day 9 PBM.

#### Development of *L.* (*S*.) *hoogstraali*

**Fig. 5:**
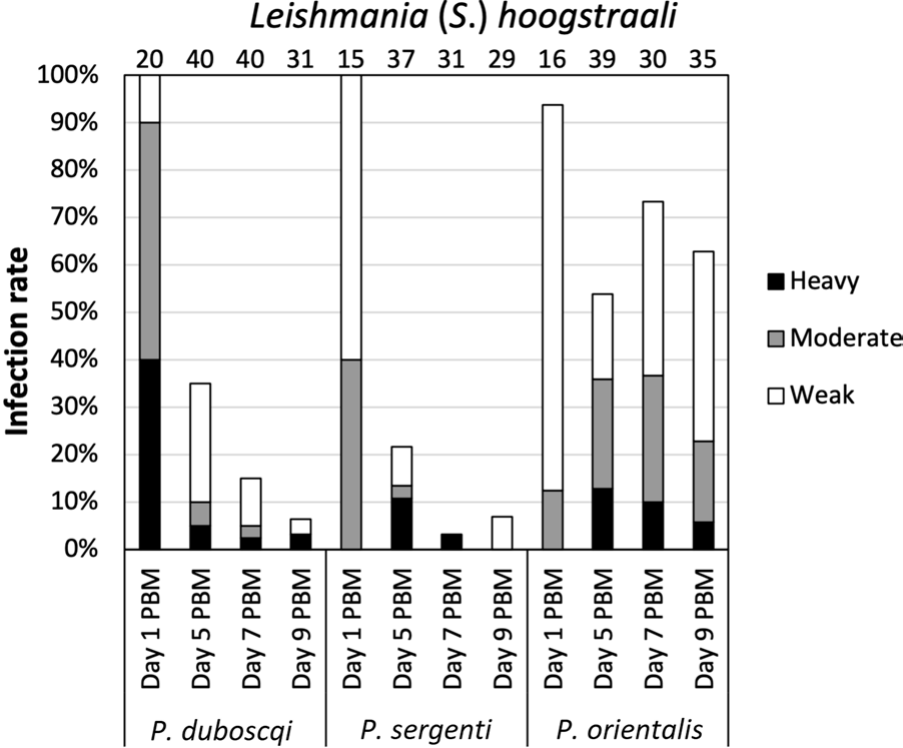
Development of *L.* (*S.*) *hoogstraali* in three sand fly species: *P. duboscqi*, *P. sergenti* and *P. orientalis*. Infection rates were examined on days 1, 5, 7 and 9 PBM. The intensities of infections were categorized as heavy (> 1000 parasites/gut), moderate (100–1000 parasites/gut) or weak/light (< 100 parasites/gut). Number of dissected sand flies is indicated above the bars

On day 1 PBM, high infection rates (94–100%) were reported in all three sand fly species, with no significant differences (*χ*^2^ = 2.231, *df* = 2, *P* = 0.328; Fig. 5). The intensities of infections were mostly weak/light or moderate, and parasites were present in the blood meal enclosed by peritrophic matrix (endoperitrophic space). After defecation, however, significant differences in infection rates were observed between sand fly species at all designated time intervals: day 5 PBM (*χ*^2^ = 8.564, *df* = 2, *P* = 0.014), day 7 PBM (*χ*^2^ = 46.269, *df* = 2, *P* ≤ 0.001) and day 9 PBM (*χ*^2^ = 35.113, *df* = 2, *P* ≤ 0.001).

**Fig. 6:**
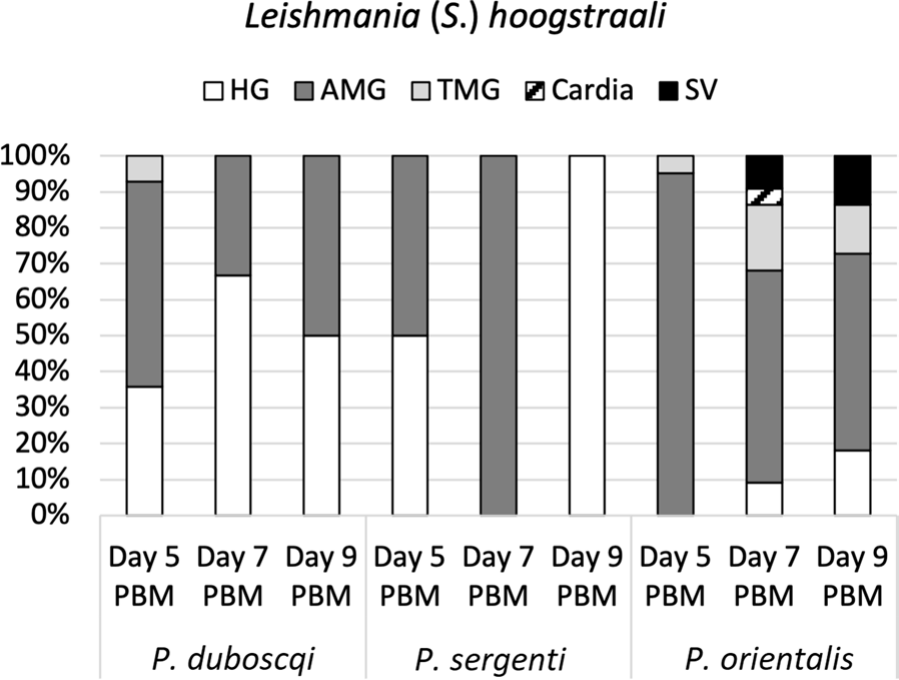
Simplified localization of *L.* (*S.*) *hoogstraali* in three sand fly species: *P. duboscqi, P. sergenti* and *P. orientalis*. Localization of infections was evaluated microscopically on days 5, 7 and 9 PBM. For abbreviations, see Fig. 2. For more details, see Additional file 2: Table S4

In *P. duboscqi* and *P. sergenti* females, *L*. (*S*.) *hoogstraali* was not able to survive defecation. The number of positive sand fly females decreased over time, and only infections of weak intensity were observed. *Leishmania* (*S*.) *hoogstraali* migrated anteriorly in *P. duboscqi*: parasites colonized mainly the hindgut but were also present in the abdominal (57%) and thoracic (7%) midgut on day 5 PBM (Fig. 6). In contrast, *L*. (*S*.) *hoogstraali* development in *P. sergenti* was restricted to the hindgut (Table 2), and promastigotes were observed in the abdominal midgut only when the remnants of ingested blood were still present.

**Table 2:** Differences in *L.* (*S.*) *adleri* and *L.* (*S*) *hoogstraali* development in various sand fly species

*Leishmania* species^a^	Sand fly species^b^					
	SCHW	PAP	ARG	DUB	SER	ORI
ADL	Hypopylarian	Hypopylarian	Peripylarian	Peripylarian	Hypopylarian	Peripylarian
HOOG	Hypopylarian	Peripylarian	Peripylarian	Peripylarian	Hypopylarian	Peripylarian

^a^*ADL* *Leishmania* (*S*.) *adleri*, *HOOG* *Leishmania* (*S*.) *hoogstraali*

^b^*SCHW* *Sergentomyia schwetzi*, *PAP* *Phlebotomus papatasi*, *ARG* *Phlebotomus argentipes*, *DUB* *Phlebotomus duboscqi*, *SER* *Phlebotomus sergenti*, *ORI* *Phlebotomus orientalis*

*Leishmania* (*S*.) *hoogstraali* successfully survived defecation and developed late-stage infections in *P. orientalis*, with infection rates reaching > than 50% at all designated time intervals. Both attached haptomonad promastigotes and free-swimming flagellates were observed in the hindgut, but *L*. (*S*.) *hoogstraali* more tended to acquire an anterior position in this sand fly species: promastigotes reached the cardia (5%) and colonized the stomodeal valve (10%) on day 7 PBM. A similar tendency was observed on day 9 PBM, when colonization of the stomodeal valve had occurred in 14% of dissected females.

### Morphological transformations

**Fig. 7:**
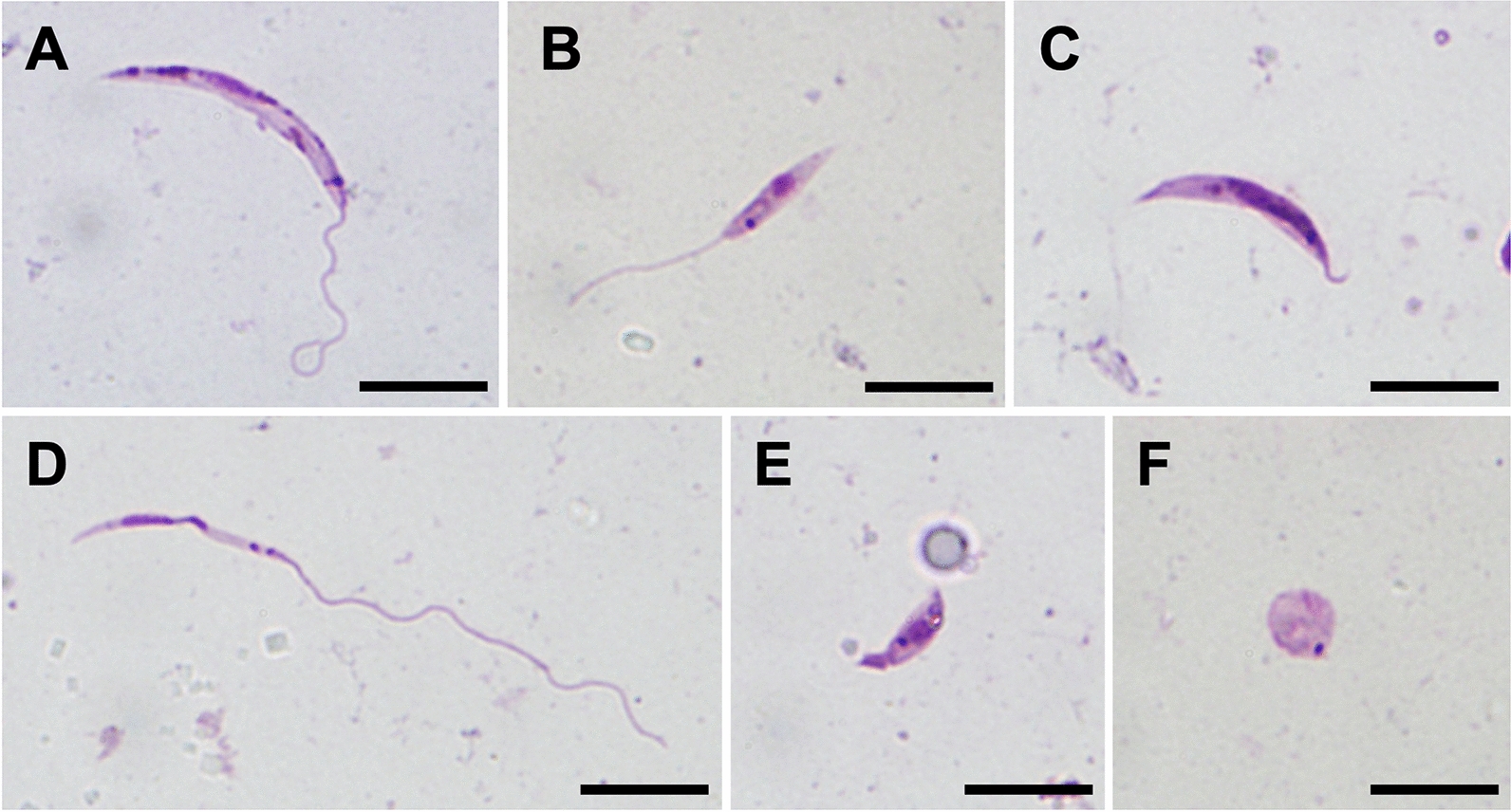
*Sauroleishmania* morphological forms in sand flies. Morphological analysis was performed on *L.* (*S.*) *adleri* and *L.* (*S.*) *hoogstraali* developing in *P. orientalis* on days 5, 7 and 9 PBM. **a** Long nectomonad promastigote, **b** short nectomonad promastigote, **c** long nectomonad promastigote with shortened flagella, **d** long slender metacyclic-like promastigote, **e** haptomonad promastigote, **f** amastigote-like form (stained by Giemsa, 1000× magnification, scale bars: 10 µm)

**Fig. 8:**
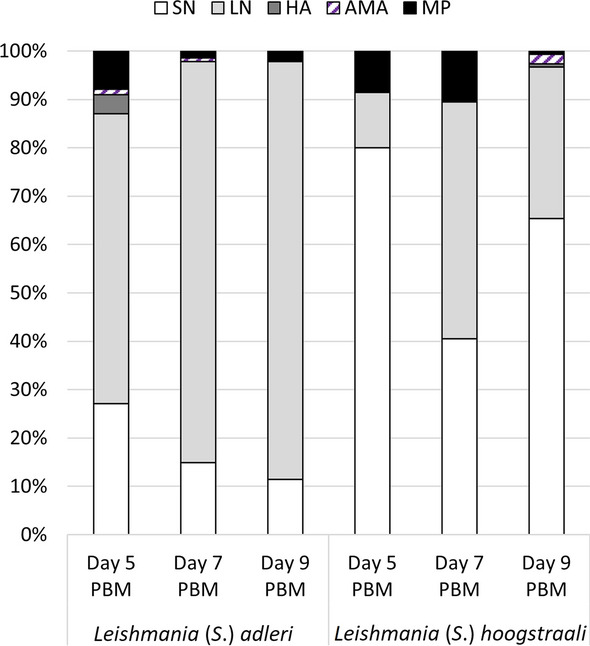
Representation of *L.* (*S.*) *adleri* and *L.* (*S.*) *hoogstraali* morphological forms in *P. orientalis* on days 5, 7 and 9 PBM. Abbreviations: SN, Short nectomonad promastigotes; LN, long nectomonad promastigotes; HA, haptomonad promastigotes; AMA, amastigote-like forms; MP, metacyclic-like promastigotes

Five morphological forms were observed in both *Sauroleishmania* species tested (Fig. 7). Long nectomonad promastigotes and short nectomonad promastigotes were the most abundant forms, while haptomonad promastigotes, metacyclic-like promastigotes and amastigote-like forms were presented to a lesser extent (Additional file 3: Table S5; Additional file 3: Table S7). Long nectomonad promastigotes prevailed in *L*. (*S*.) *adleri* (75%), whereas short nectomonad promastigotes were more frequent in *L*. (*S*.) *hoogstraali* (62%) (Fig. 8). Both long and short nectomonad promastigotes were present also in a variation with significantly shortened flagella (approx. 4 μm).

Metacyclic-like promastigotes were recorded at all designated time intervals, and these stages were morphologically highly variable in terms of body length and width. We distinguished three cell types: short rounded, short slender and long slender metacyclic promastigotes. Moreover, some of these forms had remarkably elongated flagella (up to 50 μm). Statistical analysis showed that the number of metacyclic-like promastigotes was significantly different on day 7 PBM (*χ*^2^ = 10.381, *df* = 1, *P* ≤ 0.001), but not on days 5 PBM (*χ*^2^ = 0.045, *df* = 1, *P* = 0.494) and 9 PBM (*χ*^2^ = 1.204, *df* = 1, *P* = 0.279).

Haptomonad promastigotes with typically leaf-shaped flagella were harder to detect as they are strongly attached to the cuticular lining of the sand fly gut and, therefore, their number is significantly underestimated. Rounded (amastigote-like) forms with very short or no flagella were also reported. Detailed measurements of individual morphological forms are summarized in Additional file 3: Table S6, Table S8.

### Experimental infections of geckos and xenodiagnoses

**Fig. 9:**
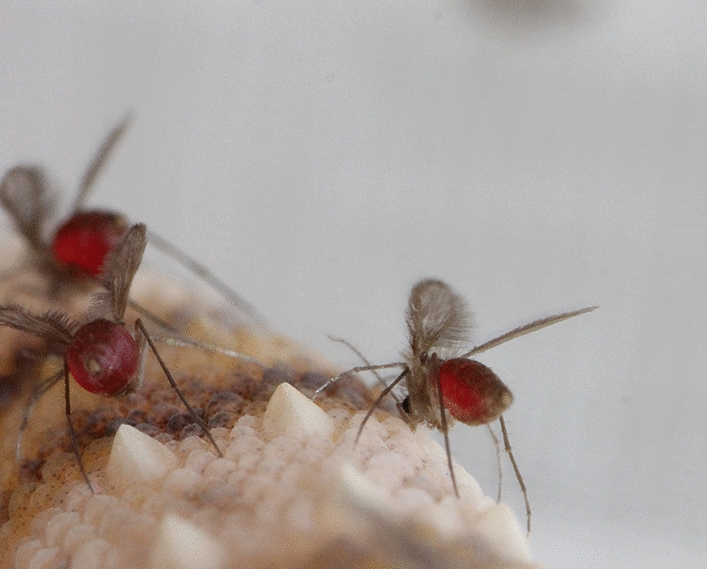
*Sergentomyia* females feeding on gecko during xenodiagnosis

No external signs of the infections were observed in any geckos. Xenodiagnostic experiments (Fig. 9) were performed on weeks 3, 7, 12 and 18 p.i., and among the 604 *Se. schwetzi* females tested, none were found to be positive (for more details see Additional file 4: Table S9). The experiment was terminated 21 weeks p.i. when geckos were sacrificed and dissected for tissue sampling. Nevertheless, *Sauroleishmania* DNA was not detected in any of the samples tested (i.e. liver, skin, tail, feet, and blood) and no parasites were observed in tissue cultures.

## Discussion

In the present study we demonstrated that the ability to undergo different types of development in sand flies is typical for parasites of the subgenus *Sauroleishmania* and that this variability is influenced by sand fly vectors. It is interesting to note that none of the parasite-vector combinations tested showed suprapylarian development and that there was always hindgut involvement to varying degrees, indicating this may be a fundamental property of *L*. (*Sauroleishmania*) species.

Although it is generally accepted that *Sauroleishmania* parasites are transmitted by reptile-biting sand flies of the genus *Sergentomyia*, the role of other sand flies in *Sauroleishmania* transmission should be reconsidered. The susceptibility of *Phlebotomus* species to *Sauroleishmania* infections has been experimentally demonstrated by several authors [13, 14] and now confirmed in the present study. Some *Phlebotomus* species were reported to feed on reptiles [4, 13, 27], and recent molecular detection of *Leishmania* (*Sauroleishmania*) *tarentolae* in *Phlebotomus* spp. [28–30] further supports the hypothesis that these sand flies are alternative vectors of *Sauroleishmania* [14].

It has been assumed that *Sauroleishmania* development in sand flies is restricted to the hindgut and described as hypopylarian [16]. Therefore, infection per the oral route was considered as one of the possible modes of *Sauroleishmania* transmission to reptiles [6]. Conversely, some older studies reported *Sauroleishmania* promastigotes in the anterior midgut [11, 13, 31]. The tendency to obtain an anterior position in the sand fly gut suggests that members of this group may be transmitted via sand fly bites, in a manner similar to mammal-infecting *Leishmania* species [15]. Nonetheless, a recent study showed that *L*. (*S*.) *tarentolae* underwent both hypopylarian or peripylarian type of development depending on the sand fly species infected [14]; consequently, the mechanism of *Sauroleishmania* transmission from sand flies to reptilian hosts remains unclear.

Despite the proven role of members of the genus *Sergentomyia* as vectors of *Sauroleishmania*, the involvement of *Se. schwetzi* in the transmission of *L*. (*S*.) *adleri* is unlikely as only 20% of females displayed the presence of parasites on day 7 PBM, with majority of infections being of weak/light intensity. It was also shown that *Se. schwetzi* is refractory to mammalian *Leishmania* spp. due to its delayed degradation of peritrophic matrix until the time of defecation, which does not provide sufficient time for promastigotes to escape the endoperitrophic space and attach to the midgut epithelium [19, 32].

Attachment of promastigotes to the sand fly gut is a key part of the *Leishmania* life-cycle as it prevents the expulsion of parasites during defecation [33]. The successful development of *L*. (*S*.) *adleri* in the hindgut of *P. papatasi* and *P. sergenti* may be due to the parasite’s ability to attach to the cuticular lining of the hindgut but its inability to bind to the sand fly midgut. *Phlebotomus sergenti* is known to be a specific vector of *Leishmania tropica* [34], while *P. papatasi* is specific for *L. major* [20] and *Leishmania turanica* [35]. In specific vectors, the attachment of promastigotes to the midgut epithelium is mediated by species-specific surface lipophosphoglycan (LPG) [36]. Nevertheless, the role of LPG in the *Sauroleishmania* life-cycle is understudied and it has been reported that some *Sauroleishmania* spp. appear to lack LPG or certain enzymes involved in LPG modification [37, 38].

Conversely, *P. argentipes* and *P. orientalis* are known to be permissive vectors susceptible to multiple *Leishmania* spp. under laboratory conditions [33] in which promastigotes attach via a different, glycan-mediated, mechanism [39]. In both of these sand fly species, the highest infection rates and highest intensities of infections were recorded for *L*. (*S*.) *adleri* and *L*. (*S*.) *hoogstraali,* suggesting that some species of *Sauroleishmania* may non-specifically attach to the midgut of permissive vectors in a manner similar to mammalian *Leishmania*.

As *Sauroleishmania* transmission from sand flies to reptilian hosts has never been demonstrated under laboratory conditions, stages infectious for the reptiles are not known [15]. Only a few studies have described *Sauroleishmania* morphological forms produced in vectors [13, 14], assuming they do not differ from those described for mammalian *Leishmania*. In this study, we demonstrated the presence of stages morphologically identical to metacyclic promastigotes. Nevertheless, the metacyclogenesis of *Sauroleishmania* has not been studied and thus the potential infectiousness of these forms is unclear.

Although sand fly-derived parasites were used for the experimental infections of geckos, *Sauroleishmania* infection was not detected in any of the *H. turcicus* tested. Selection of the wrong host species is unlikely, as *L*. (*S*.) *hoogstraali* was primarily isolated from *H. turcicus* geckos and this species has also been shown to be susceptible to *L*. (*S*.) *adleri* [18]. Therefore, we assumed that one of the possible explanations of unsuccessful transmission may be the loss of infectivity of both *Sauroleishmania* strains. Most *Sauroleishmania* isolates were obtained decades ago and have since been passaged for long periods in media without the opportunity to undergo a complete life-cycle. It has been shown that prolonged cultivation results in genetic drift and noticeable changes in the mitochondrial genome [40] and, therefore, we consider it necessary to acquire new isolates for future research work.

## Conclusions

This study provides experimental evidence that *Sauroleishmania* development in vectors is variable and significantly affected by sand fly species. Some *Phlebotomus* species, particularly *P. orientalis* and *P. argentipes*, are highly susceptible to *Sauroleishmania* infections and, therefore, the role of these sand flies in *Sauroleishmania* circulation should be reconsidered and further investigated. We also demonstrated the anterior migration of *Sauroleishmania* in their intestinal tract and confirmed the peripylarian type of development reported by several old studies.

## Supplementary Information

**Additional file 1: Table S1**. Localization of *Leishmania *(*Sauroleishmania*) *adleri *promastigotes in three sand fly species differing in vector competence to *Leishmania*. **Table S2**. Localization of *Leishmania *(*Sauroleishmania*) *hoogstraali *promastigotes in three sand fly species differing in vector competence to *Leishmania*

**Additional file 2: Table S3**. Development of *Leishmania *(*Sauroleishmania*) *adleri *in three sand fly species sharing an overlapping geographical distribution. **Table S4**. Development of *Leishmania *(*Sauroleishmania*) *hoogstraali *in three sand fly species sharing an overlapping geographical distribution

**Additional file 3: Table S5**. Representation of individual morphological forms of *Leishmania *(*Sauroleishmania*) *adleri *developing in *Phlebotomus orientalis *on days 5 to 9 post blood meal. **Table S6**. Detailed measurements of individual forms of *Leishmania *(*Sauroleishmania*) *adleri *developing in *Phlebotomus orientalis *on days 5, 7 and 9 post blood meal. **Table S7**. Representation of individual morphological forms of *Leishmania *(*Sauroleishmania*) *hoogstraali *developing in *Phlebotomus orientalis *on days 5 to 9 post blood meal. **Table S8**. Detailed measurements of individual forms of *Leishmania *(*Sauroleishmania*) *hoogstraali *developing in *Phlebotomus orient*alis on days 5, 7 and 9 post blood meal

**Additional file 4: Table S9**. Xenodiagnoses of *Hemidactylus turcicus *geckos experimentally infected with *Leishmania *(*Sauroleishmania*) *adleri *and *Leishmania *(*Sauroleishmania*) *hoogstraali*

## Data Availability

All the data are included within the article and its additional files.

## Abbreviations

ITS1: Internal transcribed spacer 1
LPG: Lipophosphoglycan
PBM: Post-blood meal
p.i.: Post-infection

## References

[1] Becvar T, Vojtkova B, Siriyasatien P, Votypka J, Modry D, Jahn P (2021). Experimental transmission of *Leishmania* (*Mundinia*) parasites by biting midges (Diptera: Ceratopogonidae). PLoS Pathog.

[2] Espinosa OA, Serrano MG, Camargo EP, Teixeira MMG, Shaw JJ (2016). An appraisal of the taxonomy and nomenclature of trypanosomatids presently classified as *Leishmania* and *Endotrypanum*. Parasitology.

[3] Akhoundi M, Kuhls K, Cannet A, Votypka J, Marty P, Delaunay P, Sereno D (2016). A historical overview of the classification, evolution, and dispersion of *Leishmania* parasites and sandflies. PLoS Negl Trop Dis.

[4] Belova EM (1971). Reptiles and their importance in the epidemiology of leishmaniasis. Bull World Health Organ.

[5] Wilson VCLC, Southgate BA, Lumsden WHR, Evans DA (1979). Lizard *Leishmania*. Biology of the Kinetoplastida.

[6] Killick-Kendrick R, Lainson R, Rioux JA, Saf'janova VM, Rioux JA (1986). *Leishmania*: Taxonomie et Phylogenèse.

[7] Rioux JA, Knoepfler LP, Martini A, Callot J, Kremer M (1969). Présence en France de *Leishmania tarentolae* Wenyon, 1921 Parasite du gecko Tarentola mauritanica (L 1758). Ann Parasitol Hum Comp.

[8] Edeson JFB, Himo J (1973). Leishmania sp in the blood of a lizard (Agama stellio) from Lebanon. Trans R Soc Trop Med Hyg.

[9] Telford SR (1979). Evolutionary implications of *Leishmania* amastigotes in circulating blood cells of lizards. Parasitology.

[10] Paperna I, Boulard Y, Hering-Hagenbeck SH, Landau I (2001). Description and ultrastructure of Leishmania zuckermani n sp amastigotes detected within the erythrocytes of the South African gecko Pachydactylus turneri Gray, 1864. Parasite.

[11] Telford SR (2009). Hemoparasites of the Reptilia.

[12] Minter DM, Wijers DJB (1963). Studies on the Vector of Kala-Azar in Kenya: IV experimental evidence. Ann Trop Med Parasitol.

[13] Adler S, Theodor O. Observations on* Leishmania ceramodactyli* N.SP. Trans R Soc Trop Med Hyg. 1929;22:343–55.

[14] Ticha L, Kykalova B, Sadlova J, Gramiccia M, Gradoni L, Volf P (2021). Development of various *Leishmania* (*Sauroleishmania*) *tarentolae* strains in three Phlebotomus species. Microorganisms.

[15] Bates PA (2007). Transmission of *Leishmania* metacyclic promastigotes by phlebotomine sand flies. Int J Parasitol.

[16] Lainson R, Shaw JJ, Peters W, Killick-Kendrick R (1987). Evolution, classification and geographical distribution. The leishmaniases in biology and medicine.

[17] Heisch RB (1958). On Leishmania adleri sp nov from lacertid lizards (Latastia sp) in Kenya. Ann Trop Med Parasitol.

[18] McMillan B. Leishmaniasis in the Sudan Republic. 22.* Leishmania hoogstraali* sp n in the gecko. J Parasitol. 1965;51:336–9. 10.2307/3275947. 5891518

[19] Sadlova J, Dvorak V, Seblova V, Warburg A, Votypka J, Volf P (2013). *Sergentomyia schwetzi* is not a competent vector for *Leishmania donovani* and other *Leishmania* species pathogenic to humans. Parasit Vectors.

[20] Pimenta PFP, Saraiva EMB, Rowton E, Modi GB, Garraway LA, Beverley SM (1994). Evidence that the vectorial competence of phlebotomine sand flies for different species of Leishmania is controlled by structural polymorphisms in the surface lipophosphoglycan. Proc Natl Acad Sci USA.

[21] Maroli M, Feliciangeli MD, Bichaud L, Charrel RN, Gradoni L (2013). Phlebotomine sandflies and the spreading of leishmaniases and other diseases of public health concern. Med Vet Entomol.

[22] Volf P, Volfova V (2011). Establishment and maintenance of sand fly colonies. J Vector Ecol.

[23] Myskova J, Votypka J, Volf P (2008). *Leishmania* in sand flies: comparison of quantitative polymerase chain reaction with other techniques to determine the intensity of infection. J Med Entomol.

[24] Sadlova J, Seblova V, Votypka J, Warburg A, Volf P (2015). Xenodiagnosis of *Leishmania donovani* in BALB/c mice using *Phlebotomus orientalis*: a new laboratory model. Parasit Vectors.

[25] Diamond LS, Herman CM (1954). Incidence of trypanosomes in the Canada goose as revealed by bone marrow culture. J Parasitol.

[26] El Tai NO, Osman OF, El Fari M, Presber W, Schönian G (2000). Genetic heterogeneity of ribosomal internal transcribed spacer in clinical samples of *Leishmania donovani* spotted on filter paper as revealed by single-strand conformation polymorphisms and sequencing. Trans R Soc Trop Med Hyg.

[27] Quate LW (1964). Phlebotomus sandflies of the Paloich area in the Sudan (Diptera, Psychodidae). J Med Entomol.

[28] Pombi M, Giacomi A, Barlozzari G, Mendoza-Roldan J, Macrì G, Otranto D (2020). Molecular detection of *Leishmania* (*Sauroleishmania*) *tarentolae* in human blood and *Leishmania* (*Leishmania*) *infantum* in *Sergentomyia minuta*: unexpected host-parasite contacts. Med Vet Entomol.

[29] Latrofa MS, Mendoza-Roldan JA, Manoj RRS, Dantas-Torres F, Otranto D (2021). A duplex real-time PCR assay for the detection and differentiation of *Leishmania infantum* and *Leishmania tarentolae* in vectors and potential reservoir hosts. Entomol Gen.

[30] Mendoza-Roldan JA, Latrofa MS, Iatta R, Manoj RRS, Panarese R, Annoscia G (2021). Detection of *Leishmania tarentolae* in lizards, sand flies and dogs in southern Italy, where *Leishmania infantum* is endemic: hindrances and opportunities. Parasit Vectors.

[31] Leishmania AS, Dawes B (1964). Advances in Parasitology.

[32] Sadlova J, Homola M, Myskova J, Jancarova M, Volf P (2018). Refractoriness of Sergentomyia schwetzi to *Leishmania* spp is mediated by the peritrophic matrix. PLoS Negl Trop Dis.

[33] Dostalova A, Volf P (2012). *Leishmania* development in sand flies: parasite-vector interactions overview. Parasit Vectors.

[34] Kamhawi S, Modi GB, Pimenta PFP, Rowton E, Sacks DL (2000). The vectorial competence of *Phlebotomus sergenti* is specific for *Leishmania tropica* and is controlled by species-specific, lipophosphoglycan-mediated midgut attachment. Parasitology.

[35] Chajbullinova A, Votypka J, Sadlova J, Kvapilova K, Seblova V, Kreisinger J (2012). The development of *Leishmania turanica* in sand flies and competition with *L* major. Parasit Vectors.

[36] Kamhawi S, Ramalho-Ortigao M, Pham VM, Kumar S, Lawyer PG, Turco SJ (2004). A role for insect galectins in parasite survival. Cell.

[37] Previato JO, Jones C, Wait R, Routier F, Saraiva E, Mendonça-Previato L (1997). *Leishmania adleri*, a lizard parasite, expresses structurally similar glycoinositolphospholipids to mammalian *Leishmania*. Glycobiology.

[38] Raymond F, Boisvert S, Roy G, Ritt JF, Legare D, Isnard A (2012). Genome sequencing of the lizard parasite *Leishmania tarentolae* reveals loss of genes associated to the intracellular stage of human pathogenic species. Nucleic Acids Res.

[39] Hall AR, Blakeman JT, Eissa AM, Chapman P, Morales-García AL, Stennett L (2020). Glycan–glycan interactions determine *Leishmania* attachment to the midgut of permissive sand fly vectors. Chem Sci.

[40] Klatt S, Simpson L, Maslov DA, Konthur Z (2019). *Leishmania tarentolae*: Taxonomic classification and its application as a promising biotechnological expression host. PLoS Negl Trop Dis.

