# Supplementary material for: Experimental infections of sand flies and geckos with Leishmania (Sauroleishmania) adleri and Leishmania (S.) hoogstraali
Source: Parasit Vectors. 2022 Aug 11;15:289. doi: 10.1186/s13071-022-05417-1 (PMC9367110; doi:10.1186/s13071-022-05417-1)
Supplement: Supplementary file 1 — Additional file 1: Table S1. Localization of Leishmania (Sauroleishmania) adleri promastigotes in three sand fly species differing in vector competence to Leishmania. Table S2. Localization of Leishmania (Sauroleishmania) hoogstraali promastigotes in three sand fly species differing in vector competence to Leishmania [file 13071_2022_5417_MOESM1_ESM.pdf]

Additional file 1:

Table S1. Localization of *Leishmania* (*Sauroleishmania*) *adleri* promastigotes in three sand fly species differing in vector competence to *Leishmania*.

		**EPS**	**HG**	**HG AMG**	**HG AMG TMG**	**HG CA**	**HG SV**	**AMG**	**AMG TMG**	**AMG CA**	**SV**
**SCHW**	D1	12									
	D7		11					1			
**PAP**	D1	19									
	D7		13					1			
**ARG**	D1	24									
	D7		4		1	1	1	12	2	1	

SCHW, *Sergentomyia schwetzi*; PAP, *Phlebotomus papatasi*; ARG, *Phlebotomus argentipes*; D1, day 1 post blood meal; D7, day 7 post blood meal; EPS, endoperitrophic space; HG, hindgut; AMG, abdominal midgut; TMG, thoracic midgut; CA, cardia; SV, stomodeal valve.

Table S2. Localization of *Leishmania* (*Sauroleishmania*) *hoogstraali* promastigotes in three sand fly species differing in vector competence to *Leishmania*.

		**EPS**	**HG**	**HG AMG**	**HG AMG TMG**	**HG CA**	**HG SV**	**AMG**	**AMG TMG**	**AMG CA**	**SV**
**SCHW**	D1	12									
	D7		2								
**PAP**	D1	19									
	D7			3					1		
**ARG**	D1	24									
	D7			2	3		2	14	7	5	6

SCHW, *Sergentomyia schwetzi*; PAP, *Phlebotomus papatasi*; ARG, *Phlebotomus argentipes*; D1, day 1 post blood meal; D7, day 7 post blood meal; EPS, endoperitrophic space; HG, hindgut; AMG, abdominal midgut; TMG, thoracic midgut; CA, cardia; SV, stomodeal valve.
