# Supplementary material for: Experimental infections of sand flies and geckos with Leishmania (Sauroleishmania) adleri and Leishmania (S.) hoogstraali
Source: Parasit Vectors. 2022 Aug 11;15:289. doi: 10.1186/s13071-022-05417-1 (PMC9367110; doi:10.1186/s13071-022-05417-1)
Supplement: Supplementary file 2 — Additional file 2: Table S3. Development of Leishmania (Sauroleishmania) adleri in three sand fly species sharing an overlapping geographical distribution. Table S4. Development of Leishmania (Sauroleishmania) hoogstraali in three sand fly species sharing an overlapping geographical distribution [file 13071_2022_5417_MOESM2_ESM.pdf]

Additional file 2

Table S3. Development of *Leishmania* (*Sauroleishmania*) *adleri* in three sand fly species sharing an overlapping geographical distribution.

		EPS	HG	HG AMG	HG AMG TMG	HG CA	HG SV	AMG	AMG TMG	AMG CA	SV
**DUB**	D1	30									
	D5		7	3	2			9			
	D7		10	4	3			10	1		
	D9		9	2				2	1		
**SER**	D1	30									
	D5		27								
	D7		42	2							
	D9		10								
**ORI**	D1	16									
	D5				4	1		3	1	1	
	D7		2	1	19	2	8	3	2	1	
	D9		4	2	2		1	4	1		

DUB, *Phlebotomus duboscqi;* SER, *Phlebotomus sergenti*; ORI, *Phlebotomus orientalis*; D1, day 1 post blood meal; D5, day 5 post blood meal; D7, day 7 post blood meal; D9, day 9 post blood meal; EPS, endoperitrophic space; HG, hindgut; AMG, abdominal midgut; TMG, thoracic midgut; CA, cardia; SV, stomodeal valve.

Table S4. Development of *Leishmania* (*Sauroleishmania*) *hoogstraali* in three sand fly species sharing an overlapping geographical distribution.

		EPS	HG	HG AMG	HG AMG TMG	HG CA	HG SV	AMG	AMG TMG	AMG CA	SV
**DUB**	D1	20									
	D5		5		1			8			
	D7		4					2			
	D9		1					1			
**SER**	D1	15									
	D5		4					4			
	D7							1			
	D9		2								
**ORI**	D1	15									
	D5			4	1			16			
	D7		2	5	2			8	2	1	2
	D9		4	4			1	8	3		2

DUB, *Phlebotomus duboscqi;* SER, *Phlebotomus sergenti*; ORI, *Phlebotomus orientalis;* D1, day 1 post blood meal; D5, day 5 post blood meal; D7, day 7 post blood meal; D9, day 9 post blood meal; EPS, endoperitrophic space; HG, hindgut; AMG, abdominal midgut; TMG, thoracic midgut; CA, cardia; SV, stomodeal valve.
