# Supplementary material for: Experimental infections of sand flies and geckos with Leishmania (Sauroleishmania) adleri and Leishmania (S.) hoogstraali
Source: Parasit Vectors. 2022 Aug 11;15:289. doi: 10.1186/s13071-022-05417-1 (PMC9367110; doi:10.1186/s13071-022-05417-1)
Supplement: Supplementary file 3 — Additional file 3: Table S5. Representation of individual morphological forms of Leishmania (Sauroleishmania) adleri developing in Phlebotomus orientalis on days 5 to 9 post blood meal. Table S6. Detailed measurements of individual forms of Leishmania (Sauroleishmania) adleri developing in Phlebotomus orientalis on days 5, 7 and 9 post blood meal. Table S7. Representation of individual morphological forms of Leishmania (Sauroleishmania) hoogstraali developing in Phlebotomus orientalis on days 5 to 9 post blood meal. Table S8. Detailed measurements of individual forms of Leishmania (Sauroleishmania) hoogstraali developing in Phlebotomus orientalis on days 5, 7 and 9 post blood meal [file 13071_2022_5417_MOESM3_ESM.pdf]

Additional file 3

Table S5. Representation of individual morphological forms of *Leishmania* (*Sauroleishmania*) *adleri* developing in *Phlebotomus orientalis* on days 5 to 9 post blood meal.

	**Day 5 PBM**	**Day 7 PBM**	**Day 9 PBM**	**Total**
**Long nectomonad promastigotes**	106	117	121	344
**Short nectomonad promastigotes**	48	21	16	85
**Metacyclic-like promastigotes**	14	2	3	19
**Haptomonad promastigotes**	7	0	0	7
**Amastigote-like forms**	2	1	0	3
**Total**	177	141	140	458

PBM, post blood meal.

Table S6. Detailed measurements of individual forms of *Leishmania* (*Sauroleishmania*) *adleri* developing in *Phlebotomus orientalis* on days 5, 7 and 9 post blood meal.

**Days PBM**	**Morphological form**	**n**	**Body length**		**Body width**		**Flagellar length**	
			**Mean (SD) (µm)**	**Range (µm)**	**Mean (SD) (µm)**	**Range (µm)**	**Mean (SD) (µm)**	**Range (µm)**
5	SN	48	11.11 (± 0.28)	5.9-14	2.15 (± 0.08)	1.2-4.5	14.43 (± 0.8)	5.5-32.1
	LN	106	18.37 (± 0.23)	14.0-25.6	1.92 (± 0.05)	1.0-4.3	20.77 (± 0.61)	3.3-38
	HA	7	8.06 (± 0.37)	6.9-9.1	2.13 (± 0.11)	1.7-2.5	2.36 (± 0.31)	1.3-4
	AMA	2	4.8 (± 0.04)	4.8-4.8	4.25 (± 0.32)	3.9-4.6	0.89 (± 0.89)	0-1.8
	MP	14	11.73 (± 1.32)	3.3-20.1	1.75 (± 0.30)	0.9-4.4	31.8 (± 3.15)	14.8-53.2
7	SN	21	13.33 (± 0.33)	8.4-14	2.31 (± 0.22)	1.4-5.1	13.59 (± 0.98)	2.8-23.5
	LN	117	18.85 (± 0.28)	14-26.8	2.1 (± 0.03)	1.4-3.1	18.38 (± 0.47)	3.8-38
	HA	0						
	AMA	1	3.79		3.88		0.87	
	MP	2	11.51 (± 5.82)	5.7-17.3	1.78 (± 0.07)	1.7-1.9	24.64 (± 10.48)	14.2-35.1
9	SN	16	12.68 (± 0.31)	9.1-13.9	2.2 (± 0.1)	1.6-2.9	13.26 (± 1.26)	4-20.9
	LN	121	18.31 (± 0.26)	14.1-29.2	2.14 (± 0.03)	1.3-3.7	17.43 (± 0.52)	3.8-34.7
	HA	0						
	AMA	0						
	MP	3	6.01 (± 0.25)	5.7-6.5	3.45 (± 1.19)	1.8-5.8	17.14 (± 2.64)	12.6-21.8

PBM, post blood meal; SN, short nectomonad promastigotes; LN, long nectomonad promastigotes; HA, haptomonad promastigotes; AMA, amastigote-like forms; MP, metacyclic-like promastigotes.

Table S7. Representation of individual morphological forms of *Leishmania* (*Sauroleishmania*) *hoogstraali* developing in *Phlebotomus orientalis* on days 5 to 9 post blood meal.

	**Day 5 PBM**	**Day 7 PBM**	**Day 9 PBM**	**Total**
**Long nectomonad promastigotes**	16	70	48	134
**Short nectomonad promastigotes**	112	58	100	270
**Metacyclic-like promastigotes**	12	15	1	28
**Haptomonad promastigotes**	0	0	1	1
**Amastigote-like forms**	0	0	3	3
**Total**	140	143	153	436

PBM, post blood meal.

Table S8. Detailed measurements of individual forms of *Leishmania* (*Sauroleishmania*) *hoogstraali* developing in *Phlebotomus orient*alis on days 5, 7 and 9 post blood meal.

**Days PBM**	**Morphological form**	**n**	**Body length**		**Body width**		**Flagellar length**	
			**Mean (SD) (µm)**	**Range (µm)**	**Mean (SD) (µm)**	**Range (µm)**	**Mean (SD) (µm)**	**Range (µm)**
5	SN	112	10.99 (± 0.16)	6.7-13.8	2.2 (± 0.04)	1.5-3.7	12.42 (± 0.5)	1-26.1
	LN	16	16.36 (± 0.58)	14-21.8	2.29 (± 0.14)	1.5-3.9	17.85 (± 1.38)	6.7-27.6
	HA	0						
	AMA	0						
	MP	12	7.27 (± 0.82)	3.9-12.8	3.3 (± 0.27)	2.1-5	19.25 (± 1.75)	9.9-29
7	SN	58	11.38 (± 0.22)	7.3-13.9	2.19 (± 0.72)	1.5-4.1	14.34 (± 0.73)	3.9-25.3
	LN	70	17.15 (± 0.36)	14-26.9	2.26 (± 0.58)	1.4-4.4	19.54 (± 0.88)	3.9-39.1
	HA	0						
	AMA	0						
	MP	15	12.03 (± 1.06)	6.9-19.7	2.58 (± 0.28)	1.8-5.9	26.81 (± 2.02)	18.1-41.8
9	SN	100	10.7 (± 0.2)	4.8-13.9	2.36 (± 0.06)	1.5-4.9	12.17 (± 0.62)	1.4-24.4
	LN	48	16.38 (± 0.27)	14.1-21.1	2.32 (± 0.07)	1.3-3.6	17.47 (± 0.63)	5.9-25.4
	HA	1	5.45		2.69		2.3	
	AMA	3	6.05 (± 0.24)	5.8-6.5	5.32 (± 0.17)	5.1-5.6	0.45 (± 0.22)	0-0.7
	MP	1	9.25		1.54		19.95	

PBM, post blood meal; SN, short nectomonad promastigotes; LN, long nectomonad promastigotes; HA, haptomonad promastigotes; AMA, amastigote-like forms; MP, metacyclic-like promastigotes.
