# Supplementary material for: Experimental infections of sand flies and geckos with Leishmania (Sauroleishmania) adleri and Leishmania (S.) hoogstraali
Source: Parasit Vectors. 2022 Aug 11;15:289. doi: 10.1186/s13071-022-05417-1 (PMC9367110; doi:10.1186/s13071-022-05417-1)
Supplement: Supplementary file 4 — Additional file 4: Table S9. Xenodiagnoses of Hemidactylus turcicus geckos experimentally infected with Leishmania (Sauroleishmania) adleri and Leishmania (Sauroleishmania) hoogstraali [file 13071_2022_5417_MOESM4_ESM.pdf]

Additional file 4

Table S9. Xenodiagnoses of *Hemidactylus turcicus* geckos experimentally infected with *Leishmania* (*Sauroleishmania*) *adleri* and *Leishmania* (*Sauroleishmania*) *hoogstraali*.

**Gecko**	**Engorged sand flies**				
	3 weeks p.i.	7 weeks p.i.	12 weeks p.i.	18 weeks p.i.	+ / -
A-I-1	5	13	11	14	-
A-I-2	12	10	14	16	-
A-I-3	17	9	5	13	-
A-P-1	21	6	12	13	-
A-P-2	14	9	13	5	-
A-P-3	19	16	10	15	-
H-I-1	28	11	14	13	-
H-I-2	9	12	10	12	-
H-I-3	10	10	9	5	-
H-P-1	12	13	7	9	-
H-P-2	30	18	10	15	-
H-P-3	19	20	6	10	-

Sand fly females were tested using conventional PCR and scored as positive (+) or negative (-). A, *Leishmania* (*S.*) *adleri*; H, *Leishmania* (*S.*) *hoogstraali*; I, intraperitoneal infection; P, peroral infection; p.i., post infection.
